# Implications of tRNA abundance on translation elongation across bovine tissues

**DOI:** 10.3389/fgene.2023.1308048

**Published:** 2023-12-19

**Authors:** Anna K. Goldkamp, Darren E. Hagen

**Affiliations:** Department of Animal and Food Sciences, Oklahoma State University, Stillwater, OK, United States

**Keywords:** tRNA, translation, bovine, ribosome profiling, gene regulation

## Abstract

**Introduction:** Translation is a crucial stage of gene expression. It may also act as an additional layer of regulation that plays an important role in gene expression and function. Highly expressed genes are believed to be codon-biased to support increased protein production, in which quickly translated codons correspond to highly abundant tRNAs. Synonymous SNPs, considered to be silent due to the degeneracy of the genetic code, may shift protein abundance and function through alterations in translational efficiency and suboptimal pairing to lowly abundant tRNAs.

**Methods:** Here, we applied Quantitative Mature tRNA sequencing (QuantM-tRNAseq) and ribosome profiling across bovine tissues in order to investigate the relationship between tRNA expression and slowed translation.

**Results:** Moreover, we have identified genes modulated at transcriptional and/or translational levels underlying tissue-specific biological processes. We have also successfully defined pausing sites that depict the regulatory information encoded within the open reading frame of transcripts, which could be related to translation rate and facilitate proper protein folding. This work offers an atlas of distinctive pausing sites across three bovine tissues, which provides an opportunity to predict codon optimality and understand tissue-specific mechanisms of regulating protein synthesis.

## Introduction

A major goal in animal genomics is to understand how changes in gene expression underlie a phenotype. Gene transcription and mRNA translation are core events in the process of gene expression. Previous research on the regulation of gene expression in livestock largely focuses on events prior to translation, including epigenetic regulation, transcription, and RNA processing. The development of strategies to quantify the transcriptome, such as RNAseq, has allowed the study of genome-wide changes in transcript abundance triggered by diverse conditions. The transcriptome gives a snapshot of transcripts expressed in a cell and is typically used as an estimate for protein abundance. However, this assumption overlooks the events that occur between transcription and translation, which may suggest a nonsynchronous relationship among transcript and protein expression. Although transcriptional control has a significant impact on the proteome, studies propose that only 40% of protein abundance can be explained by mRNA expression (de Sou et al., 2009; Maier et al., 2009; Vogel and Marcotte, 2012). One study found that certain classes of genes display high correlations between mRNA and protein expression, whereas other reports have noted discordance with a wide range of correlation coefficients (r = 0.08–0.8) (Vogel et al., 2011; Ponnala et al., 2014; Koussounadis et al., 2015; Moritz et al., 2019). This implies that mRNA measurements alone cannot account for protein level. Several studies have described the regulation that occurs during translation. During early embryogenesis in *C. elegans*, translation regulation allows the increased production of GLP-1 protein independently of transcriptional changes (Curtis et al., 1995). Valgepea et al. observed that *E. Coli* are able to achieve a higher growth rate by increasing both catalytic and translation rates of proteins (Valgepea et al., 2013). Manfrini et al. provided evidence for specific metabolic enzymes in T cells to have unchanged mRNA levels, yet increased protein abundance due to translational control (Manfrini et al., 2020). Translational control of pre-existing mRNAs facilitates a quick response to increase protein production compared to a delayed response in transcriptional regulation.

The most extensively studied factor in determining translation fidelity and efficiency is codon usage bias. Codon usage bias describes the distribution of codons across the genome, in which synonymous codons are not used in equal frequencies (Plotkin and Kudla, 2011). Sources of sequence variation, like codon usage, often drive differences in elongation rate and gene expression (Sharp and Li, 1987). Highly expressed genes are thought to be codon-biased to support efficient translation, in which the codons correspond to highly abundant tRNAs (Plotkin and Kudla, 2011; Torrent et al., 2018). Consequently, synonymous codons are under selective pressure and hybridize with tRNAs to control gene expression (Chamary et al., 2005). Although tRNAs were once thought to be ubiquitously expressed similar to housekeeping genes, dynamic differences in tRNA abundance have been observed across several tissues and diseases (Birch et al., 2016; Kirchner et al., 2017; Pinkard et al., 2020; Goldkamp et al., 2022a). Despite challenges in tRNA quantification due to tRNA redundancy and tRNA modification, recent advances in tRNA sequencing techniques have allowed improvements in detecting tRNA expression compared to tedious hybridization methods (e.g., array and Northern blotting) that cannot easily detect differences in nearly identical isoacceptors or isodecoders (Dittmar et al., 2006; Goodenbour and Pan, 2006; Fujishima and Kanai, 2014; Pinkard et al., 2020). For example, Hydro-tRNA-seq uses alkaline hydrolysis to fragment tRNAs followed by small RNA sequencing in order to avoid issues with modified tRNA bases (Gogakos et al., 2017). However, alignment of short reads can introduce difficulty in mapping. Another technique called YAMAT-seq uses a specialized Y-shaped adapter to efficiently ligate to mature tRNAs, but does not account for tRNA modifications (Shigematsu et al., 2017). A recently published protocol, Quantitative Mature tRNA sequencing (QuantM-tRNAseq), combines the use of a specialized Y-shaped and the incorporation of a demethylation treatment to survey the mature tRNA transcriptome (Pinkard et al., 2020). Our previous work investigated differential expression of tRNA genes in the skeletal muscle and liver tissue of bovine fetuses with an overgrowth syndrome (Goldkamp et al., 2022a). Through this study, we observed tissue-specific tRNA expression with dramatic changes in anticodon availability across tissues. Because a synonymous mutation could introduce a rare codon and slow translation, these findings suggest alterations in tRNA abundance could be associated with varying translation rates across tissue types. Further investigation is necessary to evaluate tRNA abundance as a source of genetic variation and the role of tRNA expression in regulating protein synthesis based on codon: anticodon interactions.

Thus far, expression studies have been directed to measure the level of all transcripts expressed in a cell, yet more work should be done to investigate the field of post-transcriptional gene regulation and its implications in livestock. In addition to their role in translation, tRNAs seem to also act as a key factor in controlling the amount of protein expressed through codon optimality. Buschauer et al. revealed instances of co-translational mRNA decay in budding yeast, in which non-optimal codons pairing to lowly abundant tRNAs triggered recruitment of the CCr4-NOT complex and resulted in decay from the 3′ end of the transcript (Buschauer et al., 2020). Indeed, Coller et al. also found that codon optimality could be inferred by mRNA half-life and was concordant with tRNA expression data (Presnyak et al., 2015; Carneiro et al., 2019). Therefore, it is thought that transcript stability increases with codon optimality, where optimality is defined by the levels of a particular tRNA.

Although the contribution of translational regulation has been understudied in bovine, recent advancements to monitor translation has offered an opportunity to examine the impacts of the tRNA transcriptome on the regulation of protein synthesis and fine tuning of proteome composition (Ingolia et al., 2011; van Heesch et al., 2019). Notably, ribosome profiling allows the isolation of mRNA fragments bound by the ribosome, revealing codon specific ribosome occupancies and actively translated regions. Similar to transcriptome analysis, translatome profiling captures all mRNAs associated with ribosomes during protein synthesis. However, read counting for translatome profiling data reflects transcript translatability instead of mRNA abundance. Prior to the development of current ribosome profiling methods, two techniques were commonly used for isolation of RNA bound by ribosomes: affinity purification and sucrose gradient centrifugation, which includes immunoprecipitation of ribosome bound RNA to release associated RNAs and the use of a sucrose gradient to separate translational components of cellular lysate (e.g., free RNA, monosomes, polysomes), respectively (Ingolia, 2010; Ingolia et al., 2012; King and Gerber, 2016). As methods for ribosome profiling have advanced, recent techniques incorporate nuclease digestion treatment to reveal positional information of the ribosome after either a pull-down or sucrose gradient approach (Ingolia et al., 2014; Clamer et al., 2018). These techniques as well as improvements in quantifying the tRNA transcriptome has enhanced the feasibility of characterizing the profiles of core elements of translational machinery in bovine and allowed the detection of translationally regulated genes.

In this study, we utilized QuantM-tRNAseq, ribosome profiling, and RNAseq across bovine muscle, kidney, and liver tissues (3 bulls in total resulting in 3 replicates per tissue type) to establish a genome-wide, high-resolution view of the translatome. The QuantM-tRNAseq and ribosome profiling protocols yielded an average of approximately 6.4 and 2.1 million mapped reads, respectively. We must address constraints and limitations of the present study. For example, there are still challenges in tRNA sequencing in the context of redundancy of tRNA genes, modifications, and aminoacylation levels (charged vs. uncharged), which may impact our results. The use of spike-in material in future studies may also enhance sensitivity in assessing tRNA load. In addition, inhibition of translation via flash freezing and cycloheximide may not provide accurate snapshots of translation. Future work should include experiments that implement other translational inhibitors, such as Tigecycline, emetine, or anisomycin, as well as an inhibitor-free approach to verify conclusions of ribosome pausing and codon usage (Xiong et al., 2018; Sinha et al., 2020; Stoneley et al., 2022). Furthermore, it is not well understood if higher ribosome density would indicate higher translation levels of a transcript or in fact indicate slower translation speed. However, the translational control underlying specific tissues is poorly understood and to our knowledge, an integrative analysis of tRNA expression and ribosome profiling in bovine tissues has not yet been performed. Overall, we observed tissue-specific variations in tRNA expression at isoacceptor and isodecoder level, identified mRNAs associated with ribosomes, and surveyed codon-specific ribosome occupancy in the presented tissue types.

## Materials and methods

### Tissue collection and RNA extraction

All processing for subsequent tissue collection was done at the abattoir (OSU Robert M. Kerr Food and Agricultural Products Center). Muscle, kidney, and liver tissue samples were collected from 3 adult bulls (2 red angus and 1 shorthorn) after slaughter and immediately flash frozen in liquid nitrogen. Tissue samples were stored at −80°C until RNA extraction. Total RNA was extracted from tissues using RNAzol (Sigma-Aldrich) combined with the Direct-zol kit (Zymo Research). Quality and concentration of the RNA samples was assessed using the Agilent Tapestation RNA ScreenTape (Agilent).

### RNA-seq processing and alignment

Total RNA samples were sent to Beijing Genomics Institute (BGI). All libraries were constructed following the DNBSEQ eukaryotic stranded mRNA library protocol. Libraries were sequenced in paired end mode (2 × 150 bp) on the DNBSEQ platform. Quality of raw reads was assessed using FastQC version 0.11.7 and SolexaQA++ version 3.1.7 dynamictrim function was used to trim low quality raw reads (Phred <20) (Andrews, 2010; Cox et al., 2010). SolexaQA++ lengthsort function was used to remove trimmed reads less than 60 bases in length. An index of the ARS-UCD1.2 genome (GCF_002263795.1_ARS-UCD1.2_genomic) was generated with hisat2-build and hisat2 version 2.1.0 was used to align paired end reads to the genome with the -dta-cufflinks option and the parameter adjustment (--mp 6,6 –score-min L,0,-0.2) to increase specificity (Kim et al., 2019). Featurecounts was used for read count estimation with the -s 2 parameter for reversely stranded data, -p to indicate paired end data as input, -T 12 parameter to specify thread number, and -M to allow multi-mapped reads (Liao et al., 2014). Read counting was performed at a feature level with parameter -t gene for read count estimation.

### QuantM-tRNAseq library preparation

Mature tRNA library preparation from muscle, kidney, and liver was performed according to the QuantM-tRNAseq protocol (Pinkard et al., 2020). In order to deacylate mature tRNAs and remove 3′ amino acids, total RNA samples were incubated at 37°C for 45 min in deacylation buffer (final concentration of 20 mM Tris-HCl pH 9.0). To remove methyl modifications from the mature tRNAs, the deacylated total RNA was treated with demethylase using the rtStarTM tRNA-optimized First-Strand cDNA Synthesis Kit (ArrayStar) and purified with the RNA Clean & Concentrator-5 kit (Zymo Research). A 3′ adapter and DNA/RNA hybrid 5′ adapters were used to hybridize the different discriminator bases preceding the 3′ CCA tail of the tRNA: 5′-TGrGrA-3′, 5′-TGrGrT-3′, 5′-TGrGrG-3′, and 5′-TGrGrC-3′. In a 200 μL PCR tube, 1 μg of deacylated total RNA was mixed with 10 pmol of 3′ adapter and 10 pmol of 5′ adapter (2.5 pmol of each DNA/RNA 5′ adapter) and then incubated in a 9 μL reaction at 95°C for 2 min 1 μL of 10x annealing buffer (final concentration of 5 mM Tris-HCl pH 8.0, 0.5 mM EDTA, and 10 mM MgCl_2_) was added to the 9 μL reaction and incubated at 37°C for 15 min to hybridize the double stranded adapters. 10 μL of 1x reaction buffer (2 μL 10x reaction buffer, 7 μL nuclease free water, 1 μL T4 RNA ligase 2 (10 U/μL)) was added to the adapter/RNA mixture and incubated at 37°C for 60 min then 4°C for 90 min. All reactions were isopropanol precipitated with glycoblue (Thermo Fisher) and suspended in 10 μL of nuclease free water. For cDNA synthesis, 1 μL of 10 μM RT primer was added to the 10 μL of adapter ligated RNA and samples were incubated at 70°C for 2 min. Following annealing of the RT primer, cDNA synthesis was done by adding 9 μL of RT reaction (final concentration of 1x RT Buffer, 0.5 mM dNTP mix, 5 mM DTT, 2 U/μL Superase-In Rnase inhibitor, and 10 U/μL of SuperScript IV Reverse Transcriptase) to the 11 μL RT primer/ligated RNA reaction for a total volume of 20 μL. The 20 μL reaction was then incubated at 55°C for 60 min. DNA-RNA dimers were removed using 2.2 μL of 1 N NaOH and samples were incubated at 98°C for 20 min. All reactions were isopropanol precipitated with glycoblue (Thermo Fisher) and suspended in 10 μL of nuclease free water. The cDNA libraries were size separated using a 6% Novex TBE-Urea PAGE gel (Thermo Fisher) and stained with ×1 SYBR gold (Thermo Fisher) in ×1 TBE Buffer for 15 min. A size selection of 100–300 bp was performed on the gel via a UV transilluminator. The gel slices were sheared, suspended in 400 μL of DNA elution buffer (final concentration of 300 mM NaCl, 10 mM Tris pH 8.0, 1 mM EDTA), incubated at −80°C for 60 min and then incubated at room temperature on a standing rotator overnight. The cDNA was isopropanol precipitated with glycoblue and resuspended in 15 μL of nuclease free water. The 15 μL of cDNA was circularized with CircLigase (Epicentre) in a 20 μL reaction (final concentration of 1x reaction buffer, 50 μM ATP, 2.5 mM MnCl_2_, and 5 U/μL CircLigase ssDNA Ligase) using the manufacturer’s suggested conditions at 60°C for 60 min and heat-inactivated at 80°C for 20 min. The circularized cDNA was isopropanol precipitated with glycoblue and resuspended in 12.5 μL of nuclease free water. PCR amplification of the circularized cDNA was done using the NEBnext Ultra Q5 next-generation master mix (NEB) with the manufacturer’s suggested conditions and were amplified for 7 cycles. A unique index primer was used for each library sample. The amplified libraries were run on a 2% agarose gel and stained with 0.05 mg/mL ethidium bromide. A size selection of 100–250 bp was performed and the gel slices were purified using the Qiaquck gel extraction kit (Qiagen). All libraries were isopropanol precipitated, suspended in 11 μL of nuclease free water, and assessed using the high-sensitivity DNA chip on the bioanalyzer (Agilent). Libraries were pooled in equal concentrations and sequenced in single-end mode (1 × 150 bp) using the Illumina NextSeq 500 System Mid Output Kit (Illumina) by the OSU Genomics and Proteomics Center.

### Ribosome profiling sample preparation and library preparation

Ribosome footprint purification and subsequent sequencing of ribosome footprints was performed with RiboLace Module 1 and LACEseq (IMMAGINA Biotechnology). Active ribosomes were isolated using the RiboLace kit according to the manufacturer’s instructions. Flash frozen tissue samples were ground with a mortar and pestle in liquid nitrogen. The resulting powder was resuspended in 800 μL of lysis buffer (final concentration of 20 mM Tris-HCl pH 7.4, 150 mM NaCl, 5 mM MgCl_2_, 1 mM DTT, 100 μg/μL cycloheximide, 1% Triton X-100, and 25 U/mL DNase I). After centrifugation at 20,000 g for 5 min, the supernatant was transferred to a new tube and kept on ice for 20 min. Ribosome footprints were generated using a nuclease digestion and ribosome footprints were captured using functionalized Ribolace beads, which were then purified using acid phenol: chloroform according to the manufacturer’s instructions. Purified samples were run on a 15% TBE-Urea polyacrylamide gel, stained using SYBR Gold, and regions corresponding to 25 to 35 nt were size selected. The gel slices were sheared, suspended in elution buffer, incubated at −80°C for 60 min and then incubated at room temperature on a standing rotator overnight. Samples were isopropanol precipitated with glycoblue and suspended in 11 μL of TR buffer. Concentration of the RPFs was assessed using the Qubit microRNA Assay Kit (Thermo Fisher).

Library preparation of purified ribosome footprints was performed using LACEseq. 5′ phosphorylation, linker ligation, circularization, reverse transcription and PCR amplification were all done according to the manufacturer’s instructions. LACEseq library preparation involves 2 rounds of PCR amplification, in which the second round incorporates a unique dual index (UDI) (IMMAGINA) for each sample. Libraries were run on a 6% TBE polyacrylamide gel, stained with SYBR Gold, and regions approximately 200 nt in size were selected. The quality and quantity of the libraries was assessed using the high-sensitivity DNA chip on the Bioanalyzer (Agilent). Libraries were pooled and sequenced in single end mode on the Illumina NextSeq 500 System High Output kit (Illumina) by the OSU Genomics and Proteomics Center and also on the Illumina NovaSeq 6000 System S1 flow cell by the Michigan State University Genomics core lab.

### tRNA-seq processing and alignment

Quality of raw reads was assessed using FastQC version 0.11.7 (Andrews, 2010). Sequencing adaptors were trimmed using the Cutadapt version 2.10 (Martin, 2011). First, 5′ adapter sequences were removed using cutadapt -u 2 and cutadapt -g TCCAACTGGATACTGGN -e 0.2, In order to remove the 3′ CCA and adapter sequences, this was then followed by cutadapt -a CCA​GTA​TCC​AGT​TGG​AAT​T -e 0.2. The adapter trimmed reads were quality trimmed using the SolexaQA++ version 3.1.7 dynamictrim utility with a Phred cut off score of 20 (Cox et al., 2010). In an effort to include truncated reads resulting from stalling during reverse transcription, quality trimmed reads with a length of at least 15 bp were kept and were sorted using the SolexaQA++ lengthsort utility (Cox et al., 2010). High confidence mature cytoplasmic tRNA sequences in the bovine reference genome (ARS-UCD1.2) were retrieved from gtRNAdb (http://gtrnadb.ucsc.edu; bosTau9-mature-tRNAs.fa) and mitochondrial tRNA sequences were retrieved from mitotRNAdb (http://mttrna.bioinf.uni-leipzig.de). A custom tRNA reference was generated by combining cytoplasmic and mitochondrial tRNA sequences into one fasta file and collapsing identical tRNA sequences. Reads were then mapped to the reference with bowtie2 version 2.3.4.1 with the following parameters: end-to-end -D 20 -R 3 -N 0 -L 15 -I S,1,0.5 –score-min C,0,0 and only exact matches were allowed (Langme et al., 2012). Samtools version 1.6 was also used to filters reads by their mapping quality score (MAPQ ≥10) over reference tRNAs. Isodecoder-level read count tables were generated using salmon version 0.11.1 and anticodon-level read tables were generated by summing the read counts of isodecoders with identical anticodons (Patro et al., 2017).

### Ribo-seq processing and alignment

Raw sequence reads from NextSeq and NovaSeq platforms for each sample were combined. Quality of raw reads was assessed using FastQC version 0.11.7 (Andrews, 2010). The LACE-seq linker (TCT​CCT​TGC​ATA​ATC​ACC​AAC​C) was trimmed from the 3′ end with Cutadapt version 2.10 (Martin, 2011). Only reads that contained the linker and had a minimum length of 29 nt were kept (20 nt + 9 nt from 5′ and 3′ UDI). Cutadapt was used to trim the T following the 5′ UDI and proceeding the RPF. To remove ribosomal RNAs (rRNA) and tRNAs, the reads were aligned to representative rRNA sequences from NCBI and the custom tRNA reference consisting of cytoplasmic and mitochondrial sequences (described previously) using bowtie2 version 2.3.4.1. The unmapped reads were then aligned to the ARS-UCD1.2 reference transcriptome, downloaded from the UCSC genome browser (refMrna.fa; https://hgdownload.soe.ucsc.edu) using bowtie2 with adjusted parameters (--mp 3,1 –-score-min L, −0.6, −0.6, -N 1 -L 19). Reads were also mapped to the reference genome (GCF_002263795.1_ARS-UCD1.2_genomic) using hisat2 with parameters--mp 3,1 –score-min L, −0.6, −0.6. Featurecounts was used on genome mapped reads for read count estimation with the adjusted parameters (-s 1, -T 12, -M, -t gene) (Liao et al., 2014).

### tRNA differential expression analysis

Differential expression analysis was performed using DESeq2 v1.30.1 for amino acid, isoacceptor and isodecoder comparisons. The median of ratios method of normalization was used with the estimateSizeFactors function of DESeq2 and the normalization factors were assigned back to the count matrix. Pairwise comparisons between each tissue type (Kidney vs. Liver, Muscle vs. Liver, Kidney vs. Muscle) were performed. Genes with a *p*-value and Benjamini–Hochberg adjusted Wald-test *p*-value ≤0.05 were classified as significant. PCA plots were generated using the plotPCA function of DESeq2. Data was CPM normalized and ggplot2 was used for bar graph visualization. Rmisc was used to calculate descriptive statistics of the data (mean, standard deviation, standard error, and 95% confidence intervals).

### Ribosome footprint analysis

To assess the consistency between replicates, Principal Component analysis (PCA) was used on genome mapped reads and generated using an unbiased rlog transformation of the normalized gene counts via DESeq2 (rlog (dds, blind = TRUE) and the plotPCA function. In order to integrate quality control of the ribosome profiling data and examine positional information (A-site, P-site, E-site), we used an R package called riboWaltz (v1.1.0.) to generate plots of average read length distribution as well as P-site enrichment in CDS and untranslated regions. Average read length distribution, P-site enrichment in CDS, and trinucleotide periodicity were computed using the rlength_distr, region_psite, and frame_psite_length functions of Ribowaltz, respectively. Count tables of codon occupancy for all sites were generated using the codon_usage_psite function with frequency normalization and altering the site parameter to specify the desired site (“asite”, “psite”, “esite”).

Differentially expressed (DEG) and differentially translated genes (DTG) were identified using the deltaTE method and read counts generated by featurecounts from RNA-seq and Ribo-seq were used as input (Chothani et al., 2019). Both DEGs and DTGS were classified as significant with a false discovery rate (FDR) ≤ 0.05. DTGs were analyzed using the database for annotation, visualization, and integrated discovery (DAVID; https://david.ncifcrf.gov) and enriched biological processes with a *p*-value ≤0.05 were considered significant (Huang et al., 2008). Translational efficiency (TE) was calculated by taking the ratio of RPKM normalized RPF counts and RPKM normalized RNA counts for each gene in each sample. Hierarchical clustering heatmaps of A, P, and E sites were created using log2, mean centered data and the pheatmap function. Codon usage analysis was performed on DTGs that were upregulated in each tissue. The CDSs of each gene in the bovine reference genome (ARS-UCD1.2) were retrieved from Ensembl Biomart version 104. Frequency and relative synonymous codon uses (RSCUs) were calculated using the “Bio:Tools:CodonOptTable” BioPerl module and custom PERL scripts were used to average frequency and RSCU values.

## Results

### QuantM-tRNAseq allows high-throughput sequencing of mature tRNAs

We used a previously published protocol, QuantM-tRNAseq, in order to accurately quantify changes in tRNA expression levels among bovine muscle, kidney, and liver tissue (n = 3 per tissue). QuantM-tRNAseq provides an improved representation of the tRNA transcriptome through increased adapter ligation efficiency and removal of reverse transcription (RT) blocking modifications via demethylation treatment (Pinkard et al., 2020). Adapters and 3′ CCA tails were removed from raw sequenced reads followed by quality trimming (Phred ≥20), which yielded an average of 11,660,791 clean reads across all samples (Supplementary Table S1). The trimmed reads were aligned to a custom reference set of high-confidence mature tRNA sequences (ARS-UCD1.2) obtained from gtRNAdb and mitotRNAdb, where confidence was based on functional score thresholds via tRNA-ScanSE (Supplementary Table S1) (Rosen et al., 2020). Although the bovine reference genome (ARS-UCD1.2) has 1,659 annotated tRNA genes, many of these genes have identical or nearly identical sequences. Due to the limitations introduced by the genetic redundancy of tRNAs, identical tRNA sequences were collapsed into a single representative in our reference set. Therefore, our tRNA reference set included a total of 565 unique tRNA transcript sequences, representing 21 amino acids (including selenocysteine) and 54 anticodons. Only 54 out of 62 anticodons were present because 8 tRNAs (Ala-GGC, Arg-GCG, Asn-ATT, Gly-ACC, His-ATG, Leu-GAG, Pro-GGG, Tyr-ATA) were not a part of the high confidence tRNA list due to low feature scores. In other words, these tRNAs are likely non-functional in translation due to critical sequence variations or represent tRNA-derived short interspersed repeated elements (SINEs) (Chan et al., 2019). A previous report illustrates missing tRNA genes by identifying absent tRNA isoacceptors across 100 species of Bacteria, 50 species of Archaea, and 60 species of Eukarya (Ehrlich et al., 2021). In this report, Asn-ATT, Gly-ACC, His-ATG, and Tyr-ATA were absent from all species analyzed (Bacteria, Archaea, and Eukarya). Alternatively, Ala-GGC, Leu-GAG, and Pro-GGG were missing from all species of Eukarya, while Arg-GCG was absent from all Eukarya and Bacteria species. The lack of specific tRNA genes could suggest that deleterious tRNA species are removed by negative selection and wobble base pairing is sufficient for all codons to be decoded, but perhaps at an altered speed.

Out of the 565 tRNA sequences in our gene set, 543 were classified as cytoplasmic and 22 represented mitochondrial tRNAs (Supplementary Table S2). Overall, the majority of reads aligned to cytoplasmic (Cyto) tRNAs and a smaller portion to mitochondrial (Mito) tRNAs in the liver (average of 94.1% Cyto and 5.9% Mito) and muscle (average of 84.9% Cyto and 15.1% Mito) (Supplementary Figure S1). Contrastingly, mitochondrial tRNA expression contributed to over 1/3 of all expressed tRNAs in kidney (63% Cyto and 37% Mito). Among the list of organs that have the highest oxygen consumption and mitochondrial density, the kidney is only second to the heart (Bhargava and Schnellmann, 2017; O’Connor, 2006). Therefore, an increase in the proportion of mitochondrial-derived tRNAs could likely be related to the high energy demands of the kidney compared to liver and muscle.

### Tissue-specific expression of tRNAs across bovine tissues

**FIGURE 1:**
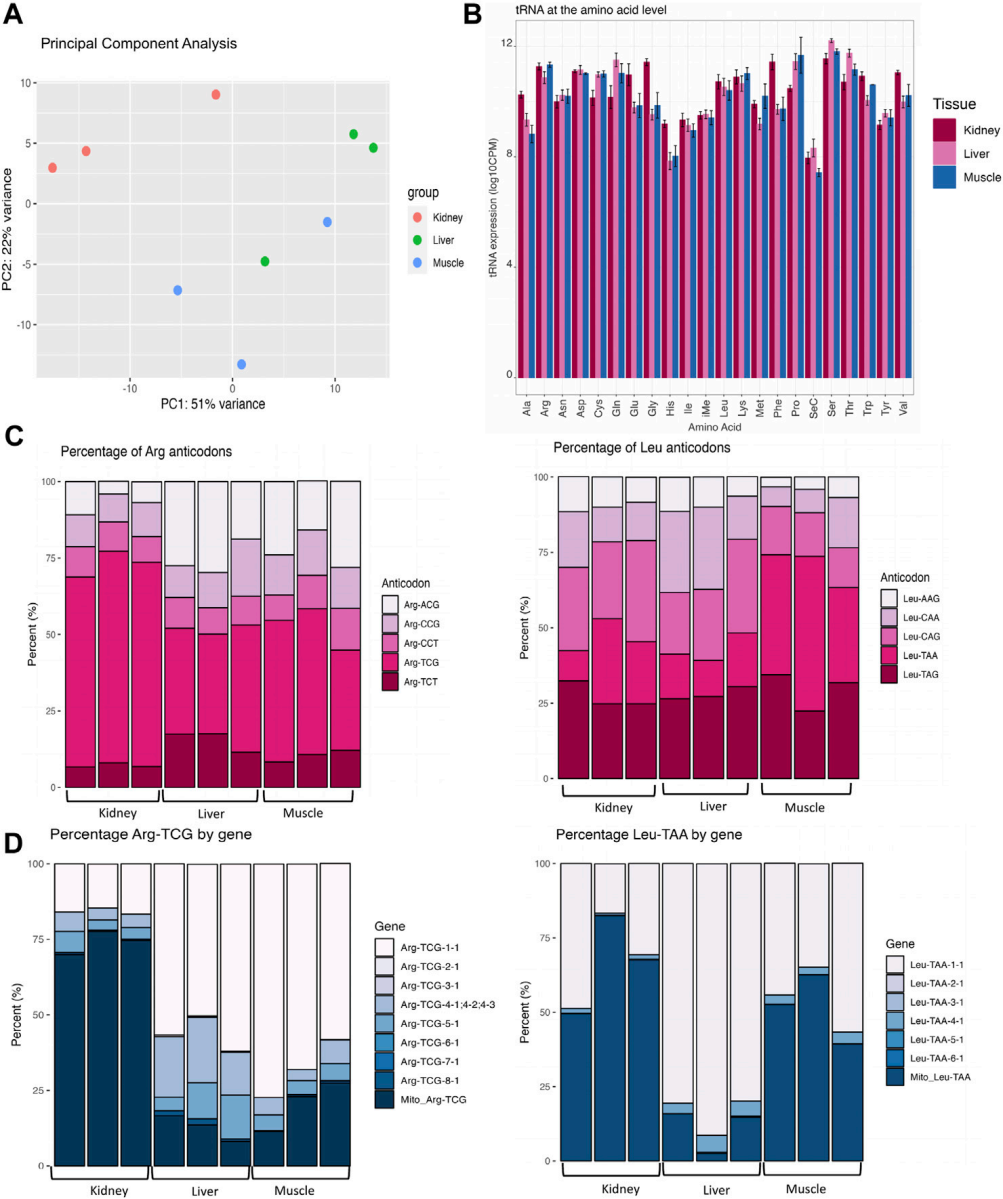
Variation in tRNA expression across bovine tissues. **(A)** Principal component analysis (PCA) reveals the similarity of expression between muscle, kidney, and liver tissues. **(B)** Bar plots displaying tRNA expression for each amino acid. Expression for each codon for all forms of isodecoder tRNAs were summed at the level of the amino acid and categorized based on tissue type. The *y*-axis represents the log10 CPM-normalized values by each amino acid and tissue type. Pooled standard error bars are shown for all anticodons within each amino acid family and were computed using the SummarySE function of the Rmisc package. **(C)** Stacked bar charts of tissue-specific variation in anticodon expression within Arginine (Arg) and Leucine (Leu). All replicates are shown for all tissue types and the *y*-axis represents the percentage of each anticodon contributing to the abundance of the amino acid. **(D)** Stacked bar charts of tissue-specific variation in isodecoder expression within Arginine-TCG and Leu-TAA. Bar charts represent the proportion of distinct isodecoders making up the pool of Arg-TCG (left) and Leu-TAA (right). Stacked bar charts represent data that was CPM-normalized and percentage of each isoacceptor/isodecoder was calculated. All isodecoders are plotted in these graphs regardless of very low (Arg-TCG-6-1 and Arg-TCG-7-1) or high (Arg-TCG-1-1) expression levels.

In the present study, we applied the QuantM-tRNAseq protocol for tRNA sequencing in an effort to increase sensitivity and capture more tRNA species (Pinkard et al., 2020). In order to reduce dimensionality and investigate the reproducibility of replicates, principal component analysis (PCA) was used on the tRNA expression data. PC1 and PC2 captured 51% and 22% of the variance respectively (Figure 1A). Biological replicates for each tissue clustered together with kidney replicates having the strongest degree of separation from muscle and liver replicates, which could perhaps be due to differences in Mito tRNA expression as previously discussed. This suggests that there is enough variation in tRNA expression to allow us to differentiate diverse tissue types. DESeq2 was used to perform a differential expression (DEG) analysis between any two tissues at the level of the amino acid, anticodon, and isodecoder. A DEG significance threshold of false discovery rate (FDR) ≤ 0.05 was used. The full DEG output for each comparison at all levels can be found in Supplementary Table S3.

### Amino acid level DEG analysis

At the amino acid level, 14 of the amino acids (Ala, Cys, Gln, Glu, Gly, His, Met, Phe, Pro, Ser, Thr, Trp, Tyr, Val) were differentially abundant in at least one of the pairwise comparisons (Figure 1B; Supplementary Table S3). In a pairwise comparison between kidney and liver, 13 amino acids (Ala, Cys, Gln, Glu, Gly, His, Phe, Pro, Ser, Thr, Trp, Tyr, and Val) were differentially expressed. In a comparison between kidney and muscle, 7 amino acids (Ala, Cys, Glu, Gly, His, Phe, Pro) were differentially abundant. Between muscle and liver, Met was significantly upregulated in the muscle.

### Anticodon and isodecoder level DEG analysis

In order to connect alterations in amino acid levels to changes in anticodon expression, we performed a DEG analysis at the level of the anticodon and found that 28 tRNA anticodons were differentially expressed in at least one comparison (Supplementary Table S3). For example, Arg-ACG was upregulated in liver and muscle compared to kidney (Figure 1C, left). In addition, Leu-CAG was downregulated in muscle compared to kidney (Figure 1C, right). We also observe clear biases in the proportion of anticodons contributing to Arg and Leu decoding. For example, the majority (average of 66%) of Arg anticodons are derived from Arg-TCG in kidney, yet there is an average contribution of 36.26% and 42.27% in muscle and liver, respectively.

While isoacceptors are groups of anticodons that encode the same amino acid, isodecoders are defined as tRNAs bearing the same anticodon with sequence differences in the body of the tRNA (outside of the anticodon loop). In our data set, 108 isodecoders were differentially expressed in at least one comparison. For instance, Arg-TCG-3-1 were downregulated in muscle and kidney compared to liver while Leu-TAA-4-1 was downregulated in kidney compared to muscle and liver (Figure 1D; Supplementary Table S3). Although isodecoders have the same anticodon and are therefore functionally equivalent in terms of translational capacity, variations in isodecoder levels have been linked to biogenesis of tRNA-derived fragments (Torres et al., 2019; Goldkamp et al., 2022b). Through processing of mature tRNAs, tissue-specific tRNA-derived fragments could be produced in order to regulate gene expression and control homeostasis. This means that tRNAs could control protein production through their availability for translation elongation and/or through their regulatory by-products that inhibit translation initiation. The gene expression heatmap of isodecoder DEGs (Supplementary Figure S2) showed clustering of the 3 replicates for each tissue and suggests that tRNAs are dynamically regulated across tissues.

### Ribosome profiling reveals mRNAs associated with ribosomes

**FIGURE 2:**
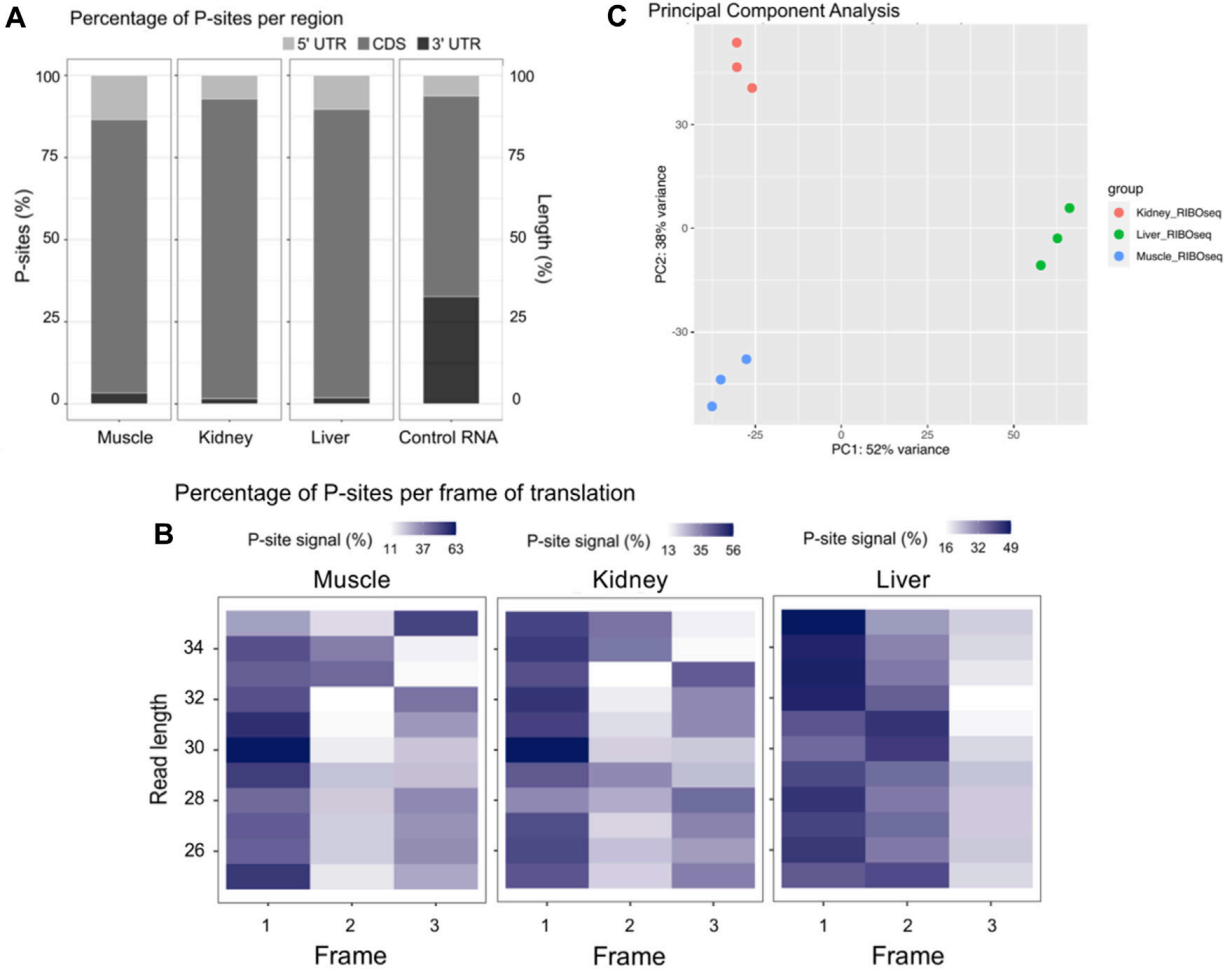
Overview of ribosome profiling data. **(A)** Representative figures for each tissue showing the percentage of P-sites in the 5′ UTR, CDS, and 3′ UTR computed with the region_psite function in the Ribowaltz package. The control RNA figure represents the expected distribution from random fragmentation of RNA. Figures for all samples can be found in Supplementary Figure S3. **(B)** Representative figures for each tissue showing the percentage of P-sites in the three frames along the CDS computed with the frame_psite_length function in the Ribowaltz package. RPFs with lengths ranging from 25 to 35 are shown. Figures for all samples can be found in Supplementary Figure S3. **(C)** Principal component analysis (PCA) of genome mapped RPFs shows tight clustering among tissue replicates and high separation across muscle, kidney, and liver.

Given the importance of tRNA availability in efficient protein synthesis and the significant diversity in their expression across tissues, we performed ribosome profiling on the same tissue samples to further characterize translational regulation. Ribowaltz was used to assess quality of the ribosome profiling data and to identify the location of the polypeptide site (P-site) within the ribosome footprints (RPFs) by calculating P-site offset (Lauria et al., 2018). The P-site is the position within the ribosome, which is bound by the tRNA holding the growing polypeptide chain during translation (Ahmed et al., 2019). The P-site offset for all samples was either 12 or 13 nt from the 5′ end of the read. In eukaryotes, RPFs are typically ∼25 to 35 nucleotides long and we found that the majority of the reads fell within this range across all three tissue types (Supplementary Figure S3A) (Ingolia, 2010; Ingolia et al., 2012). Another important characteristic in ribosome profiling data is that the majority of reads should map to the coding sequence (CDS), which is consistent with our data as the RPFs were strongly enriched in the coding sequence compared to the 5′ and 3′ untranslated region (UTR) (Figure 2A; Supplementary Figure S3B). The average percent of mapped reads falling within the CDS ranged from 85.9% to 88.6% across all tissues (Supplementary Table S6). Furthermore, the RPFs exhibited trinucleotide periodicity in the CDS, where an increased enrichment in the first frame of translation arises due to the translocation of ribosomes along each codon in an mRNA transcript (Figure 2B; Supplementary Figure S3C; Supplementary Table S7) (Ingolia et al., 2009). This is demonstrated by the high P-site signal in Frame 1 for each tissue type compared to the other two frames of translation along the CDS (Figure 2B). PCA analysis implied there were consistent measurements between biological replicates and similar ribosome density on specific transcripts, which allowed tight clustering within each tissue (Figure 2C). We must acknowledge that muscle samples often had a reduced RPF yield during library preparation and we observed a decreased number of mapped reads in this tissue, which likely impacted the number of ribosome-bound transcripts detected (Supplementary Table S4).

### Integration of RiboSeq and RNAseq reveals tissue-specific translational regulation

Because muscle, kidney, and liver tissues underlie economically important traits in livestock, such as feed efficiency and growth, we were particularly interested in tissue-specific translational regulation. In an effort to determine translationally regulated and transcriptionally regulated genes, we implemented DeltaTE analysis (Chothani et al., 2019). DeltaTE integrates matched RiboSeq and RNAseq datasets in order to detect genes with differential translational efficiency through an interaction term that models change in translational efficiency (TE). First, DeltaTE was used to predict genes with significant changes in TE (Supplementary Table S10). Another method of estimating TE is accomplished by taking the ratio of normalized RPFs over normalized mRNA counts within a particular gene, which represents the ribosome density per transcript standardized to mRNA abundance (Ingolia et al., 2009; Cottrell et al., 2017). In an effort to equate the DeltaTE model to standard TE calculation, we calculated TE (RPKM normalized RPF counts/RPKM normalized RNA counts) and compared it to the fold change of genes with differential TE. In general, genes with a high fold change had higher translational efficiency (Supplementary Figure S4), suggesting the DeltaTE model accurately predicts genes with differential TE.

**FIGURE 3:**
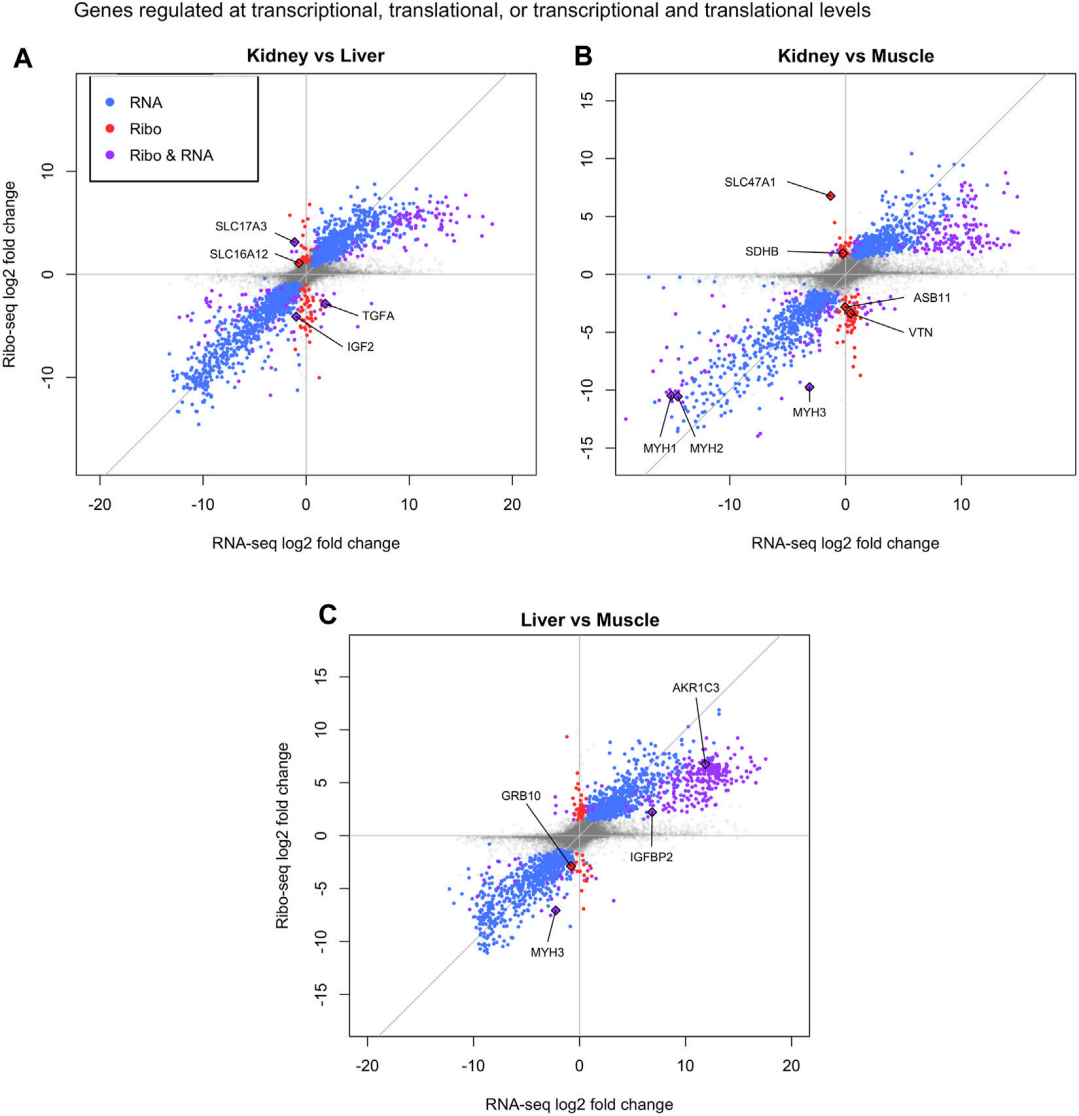
Translational regulation in bovine tissues. Differentially translated genes identified in pairwise comparisons by DeltaTE method: **(A)** Kidney vs. Liver, **(B)** Kidney vs. Muscle, and **(C)** Liver vs. Muscle. The *x* and *y*-axis are the log2FoldChange of the RNAseq and Ribosome profiling data respectively. Blue dots represent transcriptionally regulated genes and red dots indicate translationally regulated genes. Purple dots display genes regulated at both transcriptional and translational levels. Select genes underlying tissue-specific biological processes are labeled.

In addition, the DeltaTE method was used to calculate significant changes in RPF and mRNA counts in order to assign genes to three different regulatory classes: transcriptionally regulated genes (RNA), translationally regulated genes (Ribo), and genes regulated at both transcriptional and translational levels (Ribo & RNA). On average, we found that 81.63% of the overall regulatory changes were due to transcriptional regulation, 4.29% were due to translational regulation, and 14.07% were due to both transcriptional and translational regulation. When comparing kidney to liver, a total of 72 genes were translationally regulated (Ribo), whereas a total of 160 genes were regulated by transcription and translation (Ribo & RNA) (Figure 3A, left). Furthermore, we detected a total of 85 genes regulated by translation and 247 genes regulated by both transcription and translation in kidney compared to muscle (Figure 3A, middle). Finally, there were 63 translationally regulated genes, and 300 genes regulated by transcription and translation in liver compared to muscle (Figure 3A, right). The full DeltaTE output for all genes in each regulatory class can be found in Supplementary Tables S11, S12.

Although all cell types share some core biological processes for basic cellular function, we identified differentially translated genes that describe regulatory programs underlying tissue specificity. For example, *IGF2* and TGFA were transcriptionally and translationally upregulated in liver compared to kidney as well as *IGFBP2* and *AKR1C3* compared to muscle (Figures 3A, C). *IGF2* and *IGFBP2* play an important role in energy metabolism and maintenance of liver homeostasis (Wheatcroft and Kearney, 2009; Gui et al., 2021). In addition, the liver is an organ central to steroid hormone metabolism and *AKR1C3* is an isoform in the aldo-keto reductase superfamily that modulates levels of androgens, oestrogens, and progestins (Penning et al., 2000). Moreover, *TGFA* is a mitogenic factor for hepatocytes and has a role in liver regeneration (Tomiya et al., 1998). In the kidney, we identified two solute carriers (*SLC16A12* and *SLC47A1*) that were solely upregulated via translation relative to liver or muscle, and one solute carrier (*SLC17A3*) upregulated at both transcriptional and translational levels compared to liver (Figures 3A, B). Solute carriers (SLC) are a family of proteins that are responsible for the majority of absorption, distribution, and clearance of ions/organic molecules within the renal tubule (Jutabha et al., 2010; Lewis et al., 2021; Verouti et al., 2021). Furthermore, *SDHB* was translationally upregulated in the kidney compared to muscle and knockout of SDBH has been shown to inhibit the TCA cycle (Fang et al., 2021). As mentioned previously, the kidneys are highly reliant on mitochondrial function due to their energy demands, suggesting a connection between the TCA cycle and proper kidney function (Jimenez-Uribe et al., 2021). Translationally upregulated genes in the muscle compared to kidney (*ASB11* and *VTN*) and liver (*GRB10*) participate in skeletal muscle contraction and/or muscle growth (Figures 3B, C) (Dahm and Bowers, 1998; Holt et al., 2018; Ehrlich et al., 2020). Finally, three members of the myosin family of motor proteins (*MYH1*, *MYH2*, and *MYH3*) responsible for muscle contraction were upregulated at transcriptional and translational levels in muscle compared to kidney (*MYH1* and *MYH2*) and liver (*MYH3*) (Figures 3B, C) (Schiaffino et al., 2015).

**FIGURE 4:**
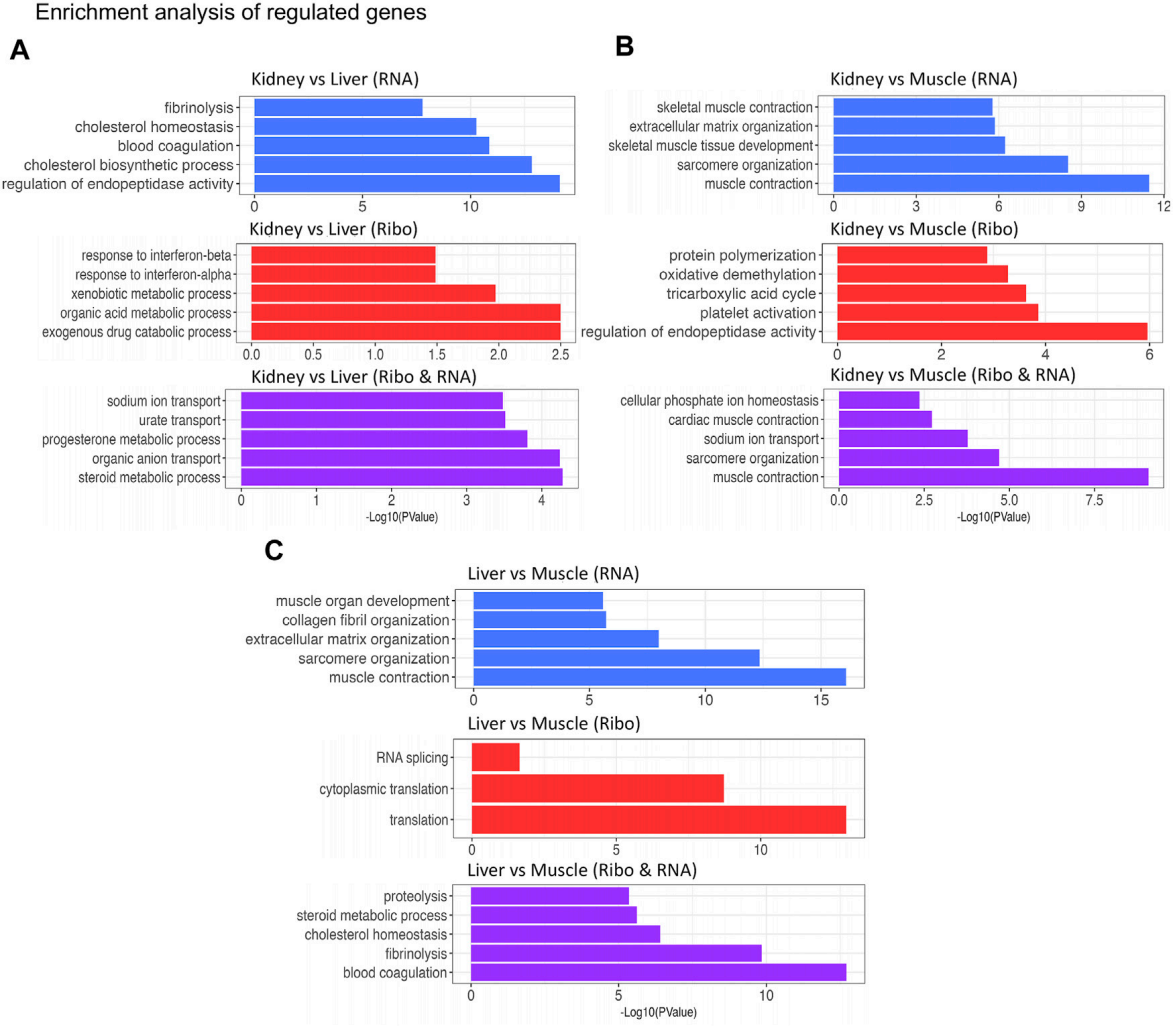
GO enrichment analysis of the different regulatory classes in each pairwise comparison: **(A)** Kidney vs. Liver, **(B)** Kidney vs. Muscle, and **(C)** Liver vs. Muscle. Graphs are colored based on class: transcriptionally regulated; RNA (blue), translationally regulated; Ribo (red), and transcriptionally and translationally regulated; Ribo & RNA (purple). Enrichment analysis was performed using DAVID and enriched pathways are ranked by *p*-value.

In an attempt to investigate the regulated genes and the processes they are involved in, GO enrichment analysis was performed for each pairwise comparison and each regulatory class (RNA, Ribo, RNA/Ribo) (Figure 4). For kidney vs. liver, differentially translated genes (Ribo; Ribo & RNA) were enriched in biological processes related to ion/metabolite transport and metabolic processes, whereas transcriptionally regulated genes were enriched in blood coagulation and cholesterol homeostasis (Figure 4A). In a pairwise comparison between kidney and muscle, genes regulated by translation were related to the TCA cycle or protein polymerization yet genes enriched in the other regulatory classes (RNA; Ribo & RNA) were enriched in muscle contraction and sarcomere organization (Figure 4B). Furthermore, transcriptionally regulated genes in liver vs. muscle were related to muscle function, while translationally regulated genes were involved in RNA splicing and translation (Ribo), and metabolism or homeostasis (Ribo & RNA) (Figure 4C). These results can help illustrate the complexities of translational regulation across diverse tissues. The full DAVID output for GO enrichment can be found in Supplementary Tables S13, S14.

### Increased ribosome pausing at specific codons within the A-site

**FIGURE 5:**
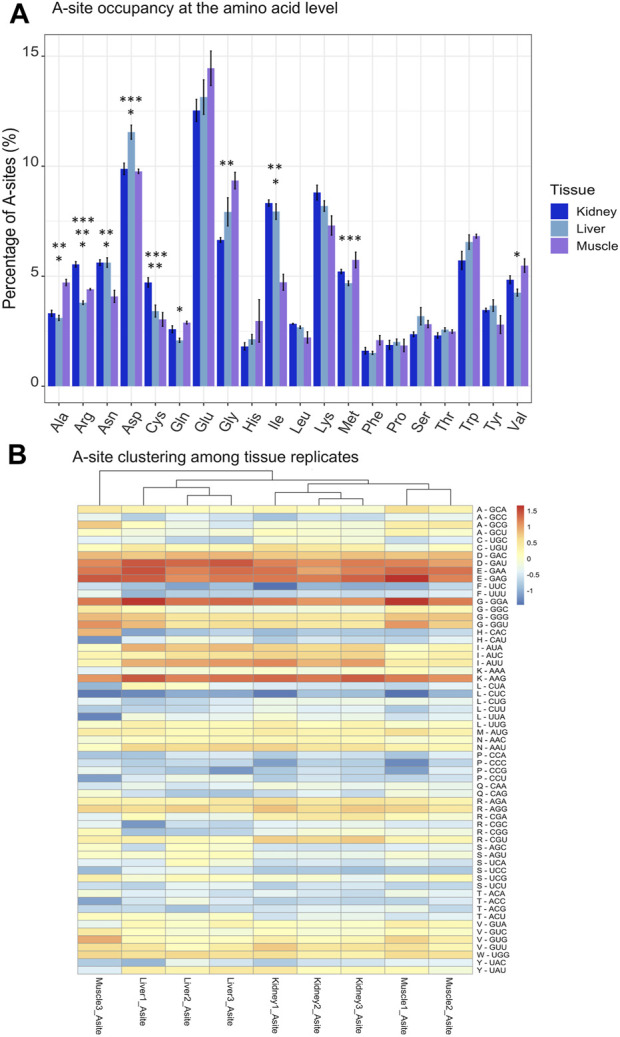
Profiles of A-site occupancy at amino acid and codon levels. **(A)** Percentage of A-sites categorized by tissue and amino acid. A-site occupancies for codons were summed based on their respective amino acid family. Standard error bars are shown for each amino acid family and were computed using the SummarySE function of Rmisc. This represents a per-molecule percentage, where A-site counts for each RPF at each codon in the transcriptome were retrieved using Ribowaltz software, normalized with the transcript frequency parameter in Ribowaltz and summed by amino acid. **(B)** Heatmap and hierarchical clustering of A-site occupancy across tissues. A-site occupancies were log2, mean centered and codons ordered by amino acid. Quickly and slowly translated codons are shown in dark blue and dark red, respectively. Asterisks indicate statistical significance after performing a Welch’s *t*-test (*p*-value ≤0.05) between Muscle and Liver (*), Kidney and Muscle (**) and Kidney and Liver (***).

After observing that tRNAs were dynamically regulated and showed high tissue specificity, we hypothesized that there could be conservation of ribosome pausing at certain aminoacyl site (A-site) codons across tissues due to variations in codon optimality. While the P-site represents the location where the tRNA is associated with the growing polypeptide chain, the A-site awaits the incoming tRNA charged with an amino acid and the exit site (E-site) holds an uncharged tRNA before its leaving during translocation (Frank et al., 2007). Ribowaltz was utilized to identify codons positioned in the A-site, P-site, and E site of RPFs, which allowed us to calculate the respective occupancy profiles for each site within the ribosome (Lauria et al., 2018). Because recruitment of the tRNA to the A-site acts as the rate limiting step during translation, we were particularly interested in A-site occupancy among tissues. The highest amount of ribosome pausing occurred at A-site codons encoding aspartic acid (Asp-D) and glutamic acid (Glu-E) (Figure 5A). Both aspartic acid and glutamic acid are negatively charged amino acids, which have previously been determined as conserved mechanisms of slowed translation across several eukaryotic species (yeast, fruit fly, zebrafish, mouse, and human) (Chyzynska et al., 2021). In addition to codons in negatively charged amino acid families, we observed specific codons with high A-site enrichment (Lys-AAG and Gly-GGA) (Figure 5B). Consecutive Lys-AAG codons within open reading frames have been observed to reduce protein production in zebrafish embryos and are suggested to trigger codon-mediated decay (Mishima et al., 2022). Although Glycine rich motifs (GGA-GGA) have been connected to translational pausing in bacteria, few reports have indicated them in eukaryotes (Rodnina, , 2016).

**FIGURE 6:**
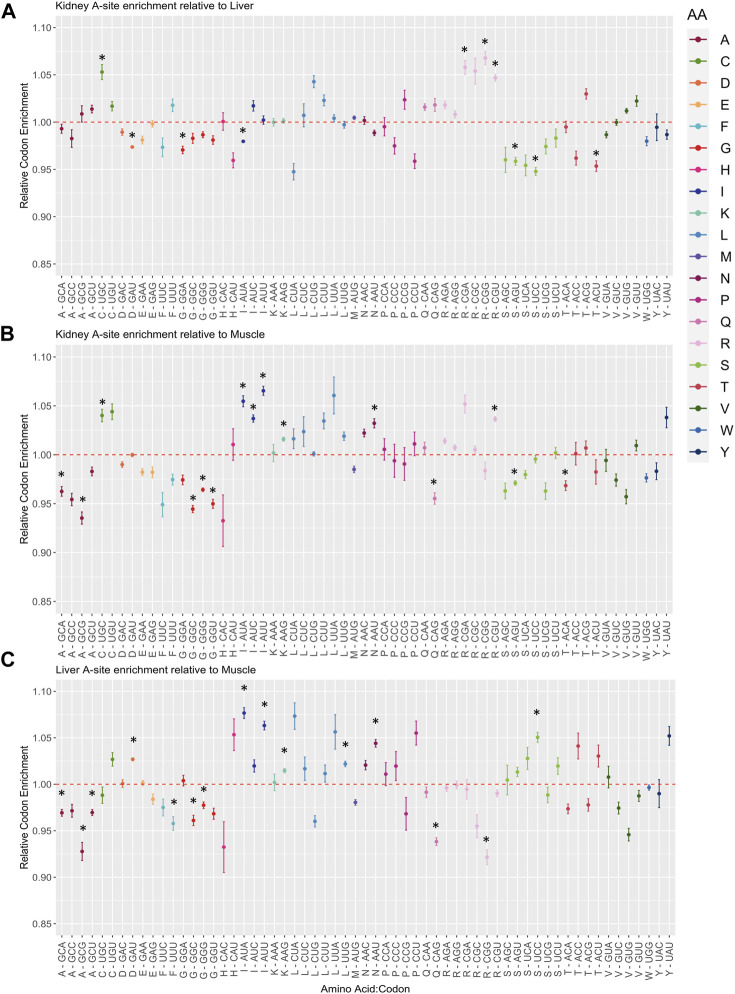
Changes in relative A-site codon enrichment depending on tissue type. **(A)** Kidney A-site enrichment relative to Liver, **(B)** Kidney A-site enrichment relative to Muscle, **(C)** Liver A-site enrichment relative to Muscle. Codons are grouped and colored by amino acid family. The average ratio of logCPM counts was calculated for each pairwise comparison by taking the logCPM ratio of all combinations of replicates between any two given tissues (Kidney #1/Liver #1, Kidney #1/Liver #2, Kidney #1/Liver #3, Kidney #2/Liver #1, *etc.*). Standard error bars were computed with the SummarySE function. Red dashed lines symbolize equal A-site enrichment for a particular codon between the two tissues compared. Codons above the red dashed line have higher enrichment in **(A,B)** Kidney and **(C)** Liver compared to other tissues. Asterisks indicate statistical significance after performing a Welch’s *t*-test (*p*-value ≤0.05).

Given the fluctuations in A-site pausing among tissues, pausing events should be conserved among biological replicates and tissues should separate from one another based off of differences in A-site codon occupancy. We found clustering among replicates for kidney and liver tissues, yet Muscle #3 was an outlier (Figure 5B). Furthermore, hierarchical clustering among A, P, and E sites for each tissue revealed that sites clustered together in kidney and liver (Supplementary Figures S5B, C). However, the A- and E-sites of Muscle #3 did not cluster with corresponding replicates (Supplementary Figure S5A). We must acknowledge that Muscle #3 had a lower yield during library preparation which resulted in a reduced number of mapped reads compared to Muscle #1 and #2, which likely resulted in it clustering away from respective replicates. Based on the first and second replicates from muscle, we observe the highest pausing at codons for Aspartic Acid (D-GAU), Glutamic Acid (E-GAA/GAG), Glycine (G-GGA), and Lysine (K-AAG) and the lowest pausing at codons for Proline (P-CCA/CCC/CCG/CCU) and Leucine (L-CUC). In the kidney and liver, we find codons can be grouped by amino acid in the P-site (Glycine (G), Leucine (L), Proline (P)), suggesting the amino acid could influence the rate of peptide bond formation. Despite variation within the muscle tissue, these findings suggest robust pausing events with tissue and amino acid specificity. To further compare changes in A-site codon occupancy, we calculated relative codon enrichment based on the average ratio of logCPM A-site occupancy counts between all tissue replicates (i.e., Kidney #1/Liver #1, Kidney #1/Liver #2, Kidney #1/Liver #3, Kidney #2/Liver #1, *etc.*) for each codon in all pairwise comparisons (Figure 6). Consistent with decreased levels of Arg-ACG in kidney, the A-site occupancy for Arg-CGU was increased in kidney compared to muscle (Figure 6B). This indicates distinctions in pausing events across tissues, which could contribute to altered levels of protein synthesis. Moreover, synonymous codons within certain amino acid families, such as Leucine, appear to have mixed effects on translation pausing (Figure 6C). These results imply that some synonymous mutations may be more or less tolerated in a given tissue. Overall, this data suggests regulatory information is encoded within the CDS through pausing mechanisms founded on codon identity, which could influence protein abundance on a tissue to tissue basis. Moreover, this data could be used as a resource for future research on the translatome and codon optimization.

### Mature tRNA expression and codon usage in differentially translated genes

After observing a distinct A-site occupancy profile in each tissue, we postulated that increased pausing at A-site codons correlated to decreased tRNA availability, and *vice versa*. Therefore, we compared A-site occupancy to tRNA expression levels in each tissue (Supplementary Figure S6). However, we did not observe a significant linear correlation in any of the tissues (*p*-value ≥0.05). Despite this, we found instances of inverse relationships between A-site codon occupancy and tRNA expression. For instance, the tRNA Asp-ATC was lowly expressed across all tissues and paired to a codon with the most A-site pausing (Supplementary Figure S6). Similarly, a Phe tRNA (Phe-GAA) was highly abundant in all tissues and coincided with a codon that was quickly translated (Supplementary Figure S6). While no clear correlation was found and the relationship between A-site occupancy and tRNA expression is quite complex, these examples illustrate a mechanism where ribosomes pause or have slowed translation at a particular codon in the A-site due to reduced availability of the cognate tRNA. We must consider that computational prediction of A-site occupancy could have introduced noise during analysis and quantification of all tRNA transcripts (charged and uncharged with an amino acid) may have presented bias. We should also acknowledge that ribosomes spend more time rejecting tRNAs than accepting them. Given that the pace of decoding during translation can be affected by the ribosome’s capacity to distinguish between perfect (cognate) and imperfect (near-cognate or noncognate) codon: tRNA pairing, the occupancy of the A-site on the ribosome might be influenced by either low levels of cognate tRNAs or an abundance of near-cognate tRNAs (Blanchet et al., 2018).

**FIGURE 7:**
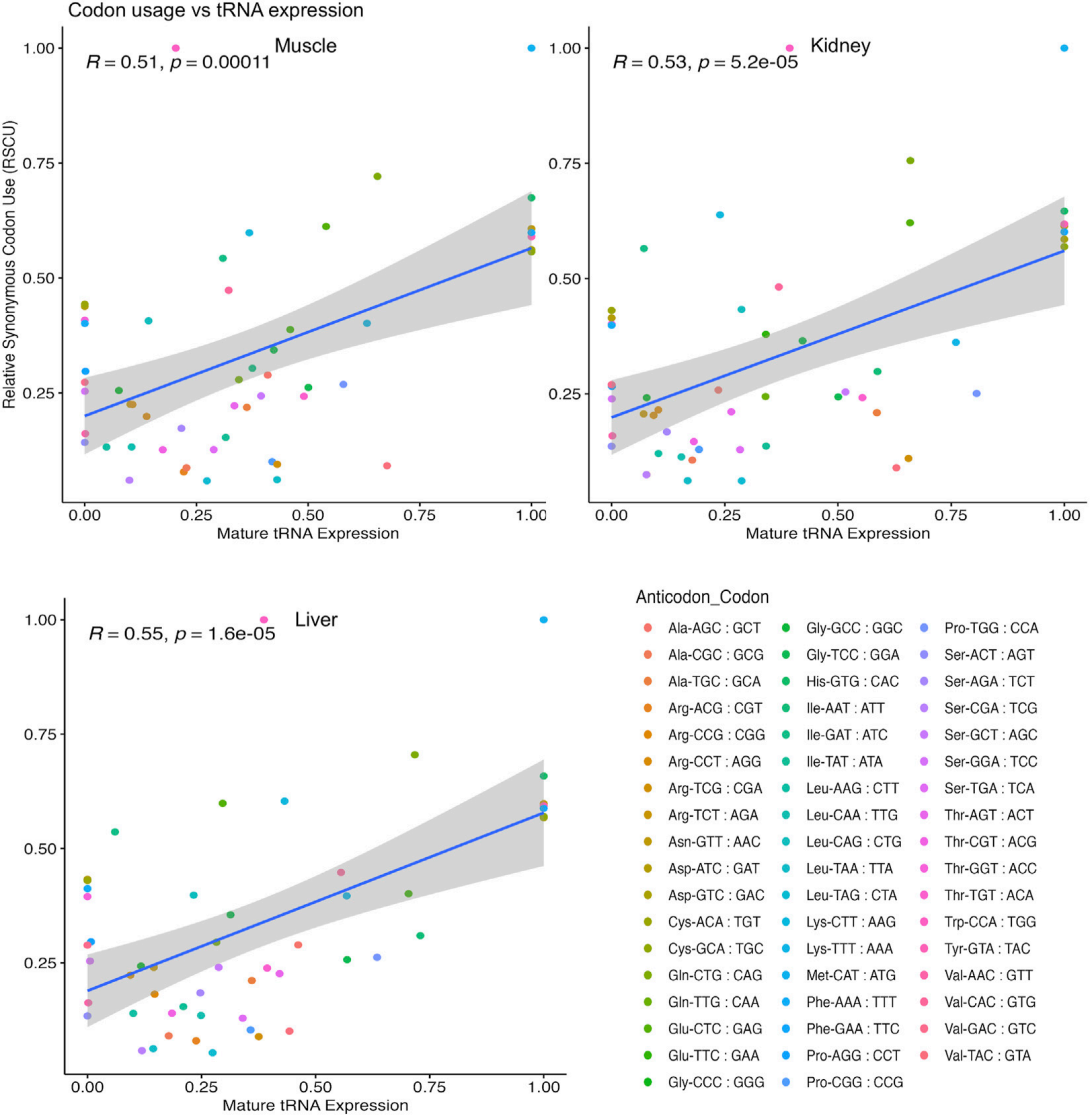
Correlation of tRNA expression and codon usage in DTGs. All isoacceptors within each amino acid family total to 100% for relative synonymous codon usage (RSCU) and tRNA expression datasets in each pairwise comparison. Test based on Pearson’s product moment correlation coefficient and regression lines were added using the geom_smooth (method = lm) function of ggplot2 to specify the linear argument method.

We next asked if alterations in tRNA abundance could be connected to differentially translated genes. Thus, we obtained the CDS of differentially translated genes in each tissue and calculated the relative synonymous codon use (RSCU) for each gene. We then performed a Pearson correlation analysis between tRNA expression and RSCU weighted by amino acid family for each tissue (Figure 7; Supplementary Table S15). Significant (*p* < 0.05) and moderately positive correlations were observed for muscle (R = 0.51), kidney (R = 0.53), and liver (R = 0.55) tissue. This observation indicates that there is some linear relationship between tRNA expression and slowed translation, and measurement of tRNA levels in bovine tissues can at least partly describe differences in translational regulation.

## Discussion

Although the redundancy of the genetic code enables multiple codons to encode the same amino acid, tRNA isoacceptors can fluctuate in their abundance across diverse cell types. While variations in tRNA expression have been described, their complexity and contribution to modulations in protein synthesis has been overlooked. In the present study, we provide a comprehensive investigation of all components of the translational machinery using QuantM-tRNAseq, ribosome profiling and RNA-seq in the first integrative analysis of translatome data in bovine tissues.

Anticodon-sparing is an interesting phenomenon that is related to decoding activity, where genomes that contain a tRNA with an A in the wobble position of the anticodon (position 34) will not also contain an isoacceptor with a G in the wobble position (Maraia and Arimbasseri, 2017). However, our dataset revealed an exception to anticodon-sparing in the cattle genome, which has a large set of tRNA genes. For example, we detected the expression of both Ile-AAT and MT-Ile-GAT with ≥50 bp reads uniquely mapping to either species with no mismatches allowed. We also observed dramatic changes in tRNA availability at amino acid, anticodon, and isodecoder levels across muscle, kidney, and liver tissue (Figure 1). Variations in the abundance of isoacceptor tRNAs could be adaptations in response to changes in transcriptome codon usage, while differential expression of isodecoder tRNAs (unique tRNAs bearing the same anticodon) could be important for the biogenesis of tRNA-derived fragments and contribute to gene regulation. Through quality control analysis with Ribowaltz, we found that our samples had characteristics of ribosome profiling data including a high enrichment of p-sites in the CDS and tri-nucleotide periodicity along the CDS (Figures 2A, B; Supplementary Figure S3).

We integrated ribosome profiling (RiboSeq) and RNA-seq datasets in order to identify both translationally and transcriptionally regulated genes via DeltaTE (Chothani et al., 2019). GO enrichment analysis revealed these translationally regulated genes were enriched in pathways related to metabolism in kidney and liver, and contraction or growth in muscle (Figure 4). These findings elucidate the contributions of both transcriptional and translational regulation in tissue-specificity. Although we observed that approximately 4.29% of regulatory changes were due to translational regulation across steady state tissues, we must recognize that there may be higher levels of translational regulation in experiments investigating alterations in cellular environment or changes in developmental state. Positional analysis via Ribowaltz allowed us to characterize codons and amino acids associated with quick and slow translation across each tissue (Figure 5; Supplementary Figure S5). Specifically, codons for glutamic acid and aspartic acid often acted as major sources of pausing across tissues, corresponding to a report that implies negatively charged amino acids act as conserved mechanisms of pausing (Chyzynska et al., 2021). Further, we find codons that are associated with quick and slow translation depending on tissue type. For example, arginine and serine codons displayed considerable variation in A-site translation rate across tissues. This indicates that translation of transcripts is discretely regulated in a given tissue due to codon composition. Some of these pause sites may contribute to proper protein folding during protein synthesis. Adjustments in pausing could also underlie quality control and trigger degradation of abnormal transcripts or peptides. These ribosome pausing profiles provide a reference to gauge codon optimality, predict translational efficiency, and estimate protein abundance in bovine.

Although translation rate and its association with tRNA expression has been well studied in unicellular organisms, these mechanisms are unclear in higher eukaryotes (Varenne et al., 1984; Dana and Tuller, 2014). Overall, we found that there was a moderately positive correlation (R = 0.51 to 0.55, *p* ≤ 0.05) between codon usage of translationally regulated genes and corresponding tRNAs among tissues (Figure 7). This implicates a role for tRNAs in fine tuning translation speed and potential for slow translated codons to be partly rescued by increased tRNA concentration. It is important to note that other factors aside from tRNA abundance could influence protein synthesis efficiency, such as tRNA modifications, amino acid sequence, mRNA sequence, and/or mRNA structure (Cannarozzi et al., 2010; Arthur et al., 2015; Boel et al., 2016; Gamble et al., 2016; Neelagandan et al., 2020). Some studies even suggest that the codon composition at the N-terminus plays an important part in stalling during elongation (Cannarozzi et al., 2010; Tuller et al., 2010; Chu et al., 2014; Verma et al., 2019). While we were able to partly connect tRNA expression to translational efficiency, we can suggest that tRNA characteristics, such as aminoacylation (charged with an amino acid), could further differentiate the availability of tRNAs for translation in a tissue specific manner. Because our study measured the expression of all tRNAs whether they were charged with an amino acid or not, this could have also introduced noise in our dataset. This work not only provides a comparative translatome analysis yielding insights into tissue-specific translational regulation, but also begins to address the correspondence between tRNA availability and translational elongation rate in diverse bovine tissues.

## Data Availability

The datasets presented in this study can be found in online repositories. The names of the repository/repositories and accession number(s) can be found below: https://www.ncbi.nlm.nih.gov/, PRJNA994961.

## Supplementary material

SUPPLEMENTARY FIGURE S1 The contribution of cytoplasmic (cyto) and mitochondrial (mito) tRNAs to the total tRNA pool. Stacked bar graphs represent the percentage of reads for each replicate that correspond to mitochondrial and cytoplasmic tRNAs.

SUPPLEMENTARY FIGURE S2 Heatmaps and hierarchical clustering of differentially expressed tRNAs at the level of the isodecoder.

SUPPLEMENTARY FIGURE S3 Quality control of ribosome profiling data. **(A)** Average read length distribution across tissues. **(B)** Percentage of P-sites in the 5′ UTR, CDS, and 3′ UTR across all replicates. **(C)** Percentage of P-sites in the three frames along the CDS in all replicates.

SUPPLEMENTARY FIGURE S4 Scatterplot illustrating the relationship between TE calculation and fold change of genes with differential translational efficiency (TE) predicted by the DeltaTE model. Red and blue dots indicate genes predicted to have high and low translational efficiency respectively.

SUPPLEMENTARY FIGURE S5 Heatmap and hierarchical clustering of A, P, and E sites within **(A)** muscle, **(B)** kidney, and **(C)** liver tissues. A-site occupancies were log2, mean centered and codons ordered by amino acid. Quickly and slowly translated codons are shown in dark blue and dark red, respectively.

SUPPLEMENTARY FIGURE S6 Relationship between A-site occupancy and Mature tRNA expression. Scatterplot showing the correlation between A-site (log2) and tRNA expression (log2) using the Pearson product moment correlation coefficient.

## Abbreviations

A-site, aminoacyl site; BGI, Beijing Genomics Institute; CPM, counts per million; CDS, coding sequence; DEG, Differentially expressed genes; DTG, Differentially translated genes; E-site, exit site; FDR, false discovery rate; MAPQ, Mapping quality score; P-site, polypeptide site; PCA, Principal Component analysis; QuantM-tRNAseq, Quantitative Mature tRNA sequencing; RPF, ribosome footprints; RT, reverse transcription; rRNA, ribosomal RNA; RSCU, Relative synonymous codon use; SINE, short interspersed repeated elements; SRA, Sequence read archive; tRNA, transfer RNA; UTR, untranslated region; UDI, unique dual index.
